# Longitudinal monitoring of circulating tumor cell phenotypic conversion as a predictor of late recurrence in breast cancer: a prospective feasibility study using the GenoCTC platform

**DOI:** 10.3389/fonc.2026.1903915

**Published:** 2026-07-31

**Authors:** Chaithanya Chelakkot, Vipin Shankar Chelakkot, Hyehyung Shin, Chaeyeol Cho, Jieun Park, Jiu Choi, Dayeong Hong, Hun Seok Lee, Young Kee Shin

**Affiliations:** 1 Research Institute of Pharmaceutical Science, Department of Pharmacy, Seoul National University College of Pharmacy, Seoul, Republic of Korea; 2 Department of Cancer Science, Cleveland Clinic Research, Cleveland, OH, United States; 3 Case comprehensive Cancer Center, Case Western Reserve University, Cleveland, OH, United States; 4 Department of Food and Nutrition, Dong-Eui University, Busan, Republic of Korea; 5 Research Institute, Genobio Corp, Seoul, Republic of Korea; 6 Department of Molecular Medicine and Biopharmaceutical Sciences, Graduate School of Convergence Science and Technology, Seoul National University, Seoul, Republic of Korea; 7 Research Institute, Cichlid Corporation, Seoul, Republic of Korea

**Keywords:** breast cancer dormancy, circulating tumor cells, c-MET proto-oncogene proteins, epithelial-mesenchymal plasticity, liquid biopsy, molecular recurrence

## Abstract

**Purpose:**

Patients with hormone receptor–positive (HR+) breast cancer faces a persistent, constant risk of distant recurrence for over 20 years. This proof-of-concept study evaluated the clinical utility of circulating tumor cell (CTCs) monitoring using the GenoCTC^®^ platform to detect molecular precursors of late recurrence in long-term breast cancer survivors.

**Methods:**

Blood samples from 25 breast cancer patients in recurrence-free survival (RFS), 4–13 years post-surgery, were analyzed using the GenoCTC^®^ platform with EpCAM and Vimentin as markers. Baseline CTCs were characterized by immunofluorescence staining (DAPI+/Cytokeratin (CK)+/CD45-). A subset of eight initially CTC-negative patients underwent longitudinal follow-up assay at a 5-year interval, with expanded profiling including c-MET enrichment. CTCs counts were correlated with clinicopathological variables, receptor status and clinical outcomes.

**Results:**

At baseline, CTCs were detected in 24% of patients. CTC burden was significantly associated with recurrence (P <.0001) and mortality (P = .0016). A high CTC count was identified in all recurrence events (P = .003). In longitudinal follow-up, 6 of 8 (75%) initially CTC-negative patients demonstrated “molecular conversion” characterized by a “burst” of epithelial-mesenchymal hybrid CTCs, including CK+/Vimentin+ and cMET+ populations.

**Conclusion:**

Longitudinal phenotypic CTC profiling could identify a pre-clinical “molecular conversion” in breast cancer survivors, which has the potential to provide a time lead over radiographic appearance of recurrence. Integration of EpCAM, Vimentin and cMET multi-marker CTC monitoring into standard long-term follow-up may enable early interception of dormancy escape and late relapse.

## Introduction

Breast cancer (BC) is the most common malignancy and the leading cause of cancer-related deaths in woman globally accounting for approximately 32% of all female cancer diagnosis (1). While significant advances in adjuvant therapy have improved early outcomes, late-recurrence, defined as relapse occurring 5 or more years following primary treatment, remains a persistent and inadequately addressed clinical challenge, particular in hormone receptor-positive (HR+) disease (2, 3).

The biology of late recurrence is fundamentally governed by tumor dormancy, whereby residual disseminated tumor cells (DTCs) persist in a quiescent state for years to decades before re-emerging as clinically detectable metastases. The Early Breast Cancer Trialists’ Collaborative Group (EBCTCG) pooled analysis of 150,000 women confirms that while 10-year recurrence risks have declined by 25% since 2000, ER+ relapse continues at a constant rate for over 20 years after diagnosis, with a 17% cumulative incidence of recurrence between 10 and 32 years reported in a Danish national database of more than 20,000 patients (3, 4). Critically, in early-stage, node-negative patients, recurrence ratios between 10 and 20 years can exceed those of the first decade, underscoring the persistence of residual microscopic disease (5).

This “dormancy paradox” highlights a gap in our oncological toolkit: despite improved adjuvant therapies, a significant subset of patients remains in unstable dormancy with a perennial risk of “awakening.” Current surveillance strategies rely primarily on clinical examination and radiographic imaging, which are inherently reactive, detecting disease only after macroscopic establishment. While genomic risk tools, including, multi-gene expression assays such as the Breast Cancer Index (BCI), the 21-gene recurrence score, and the IHC4 assay, provide valuable prognostic stratification, they remain static snapshots of a patient’s risk profile at the time of surgery (6–10). Static assays are inherently limited in their ability to monitor the dynamic, real-time “molecular recurrence” that occurs in the years following initial treatment. An ability to monitor biological transition from stable dormancy to systemic relapse is therefore an unmet and urgent clinical need.

Circulating tumor cells (CTCs); cancer cell shed into peripheral blood stream; have emerged as minimally invasive biomarkers capable of providing dynamic, real-time information about tumor behavior (11, 12). In metastatic breast cancer, elevated CTC counts are established prognostic markers for disease progression and survival (12, 13). Critically, a landmark sub-study of the E5103 trial demonstrated that the presence of even a single CTC in breast cancer patients five years or more post-diagnosis, during their RFS period, was associated with a greater than 13-fold increase in the risk of late recurrence (14). These observations strongly support a role for CTC monitoring in long-term breast cancer survivorship care.

The phenotypic characterization of CTCs adds further biological depth. EpCAM-based enrichment, the standard approach, captures epithelial CTCs, but is known to under detect cells that have undergone epithelial-mesenchymal transition (EMT), a key mechanism enabling systemic dissemination and resistance to therapy (15, 16). Vimentin, a mesenchymal marker expressed on tumor cells undergoing malignant transformation has been validated as a complementary marker for capturing EMT CTC sub-populations (17, 18). Furthermore, c-MET, a proto-oncogene receptor tyrosine kinase and hepatocyte growth factor receptor- has been identified as an independent prognostic factor in CTCs of HR+/HER2-negative metastatic breast cancer, with its expression associated poor prognosis (19).

We have previously validated the GenoCTC immune-magnetophoretic microfluidic platform as an efficient system for CTC enrichment from whole blood (19, 20). In this prospective feasibility study, we investigate whether longitudinal multi-marker phenotypic profiling could identify a biologically meaningful precursor event; which we term “molecular conversion”; preceding late recurrence in long-term breast cancer survivors. Using the GenoCTC assay (20), we report CTC burden at RFS and its correlation with recurrence. Additionally, we demonstrate that late recurrence could be preceded by a measurable biological “burst” marked by phenotypic shifts toward mesenchymal (Vimentin) and bypass signaling (c-MET) markers. This observation suggests that tracking the molecular conversion of CTCs could potentially identify patients at risk of clinical relapse before symptoms appear.

## Methods

### Patient population and study design

This prospective-retrospective study initially screened 29 patients with a history of early-stage breast cancer, and blood samples were collected during routine health screenings at IMC Gangnam clinic, Seoul, Republic of Korea, between December 2018 and February 2019. Four patients were excluded from the baseline analysis as they were <4 years post-primary treatment. The eligible cohort comprised patients in their RFS period, ranging from 4 to 13 years after initial surgery (median: 7.3 years). All patients were clinically free of disease at enrollment. A subset of patients (n=8, including 2 patients who had reached the >4-year threshold by the second timepoint underwent longitudinal surveillance with a secondary liquid biopsy at a median interval of 5 years (conducted between April 2024 and August 2024)). The study was conducted in accordance with the Declaration of Helsinki and approved by the Institutional Review Board (IRB No. 2-20181130-11350636). Written informed consent was obtained from all participants prior to enrollment.

### Peripheral blood mononuclear cell isolation

PBMCs were isolated from all samples using a density gradient centrifugation method for preparation of immunocytochemistry (ICC) control slides. Briefly, 3 mL of Lymphoprep™ (Stemcell technologies, Vancouver, Canada) was added to a 15 mL conical tube. One milliliter of blood was diluted with an equal volume of phosphate-buffer saline (PBS) and gently layered over the density gradient medium. Samples were centrifuged at 1,000 x g for 20 minutes at 4 °C without brake. The buffy coat layer was carefully transferred to a fresh tube and washed twice with PBS.

### CTC enrichment and multi-marker identification

For the baseline assay, 10 mL of peripheral blood was collected in EDTA tubes and stored at 4 °C. CTC isolation was performed within 4 hours using the GenoCTC platform (20). For each sample, 4.5 mL blood was incubated with the reagents in the EPCAM (EP) and Vimentin (Vim) CTC isolation kits (Genobio Corp, Seoul, Republic of Korea), separately for 30 minutes at room temperature. After incubation samples were loaded onto the GenoCTC^®^ device for separation of CTCs. For the longitudinal follow-up assay, samples were collected in Streck tubes (Streck Inc, NE, USA), and processed within 72 hours of collection. The CTC enrichment panel included Ep, Vim and c-MET. CTC counts were normalized to represent the number of CTCs per 7.5 mL of blood. A threshold of ≥1 CTCs per 7.5 mL was considered a positive result (14).

### Immunocytochemistry and CTC identification

Cells recovered from the GenoCTC device were centrifuged at 2000 rpm for 5 minutes and the supernatant removed, retaining 20 µL of sample for slide preparation. Cells were applied onto ICC glass slides placed on a hybridizer (Dako Colorado Inc, USA) set at 40 °C and allowed to dry. Cells were fixed with 4% paraformaldehyde containing 0.2% Tween-20 for 10 minutes, then blocked with 2% bovine serum albumin (BSA) for 10 minutes. Cells were stained with FITC-conjugated anti-cytokeratin 18 (CK18) and Alexa Fluor 594-conjugated anti-CD45 antibodies (GenoCTC^®^ Profiling kit, Genobio Corp, South Korea) for one hour, then mounted with Vectashield antifade mounting medium containing DAPI (Vector laboratories, USA). Baseline slides were imaged using Nikon Eclipse microscope, equipped with an Infinity 3 camera and analyzed using GenoAnalyzer v1.0 software (Genobio Corp, Seoul, South Korea). Follow-up slides were imaged and analyzed using the DeNovo CTC system (Bioview, Israel). Cells were defined as CTCs if they were DAPI+/Cytokeratin (CK)+/CD45-. CTCs were phenotypically classified as: (i) Epithelial-dominant: EpCAM+/CK+/Vimentin-/c-MET-; (ii) Mesenchymal/hybrid: CK+/Vimentin+; and (iii) Bypass-active: c-MET+/CK+. “Molecular conversion” was defined as a longitudinal transition from a CTC-negative baseline to the emergence of any CTC phenotype at follow-up. All slides were independently analyzed and enumerated by two trained researchers blinded to clinical outcome data, discrepancies were resolved by consensus review.

### Statistical analysis

The primary endpoint was the association between CTC-burden/phenotype and clinical recurrence. As CTC counts were non-normally distributed, continuous variables (e.g., CTC counts in recurrent vs. dormant groups) were compared using the Mann-Whitney U test. Categorical associations between CTC positivity and clinical characteristics or outcomes (recurrence, mortality) were assessed using Fisher’s exact test. Correlations between CTC presence and receptor status were evaluated using Pearson’s correlation co-efficient ^®^ and chi-square tests. All statistical tests were two-sided, and P <.05 was considered significant. Analyses were performed using R (version 4.2.2) and RStudio.

## Results

### Patient characteristics and baseline CTC detection

**Figure 1:**
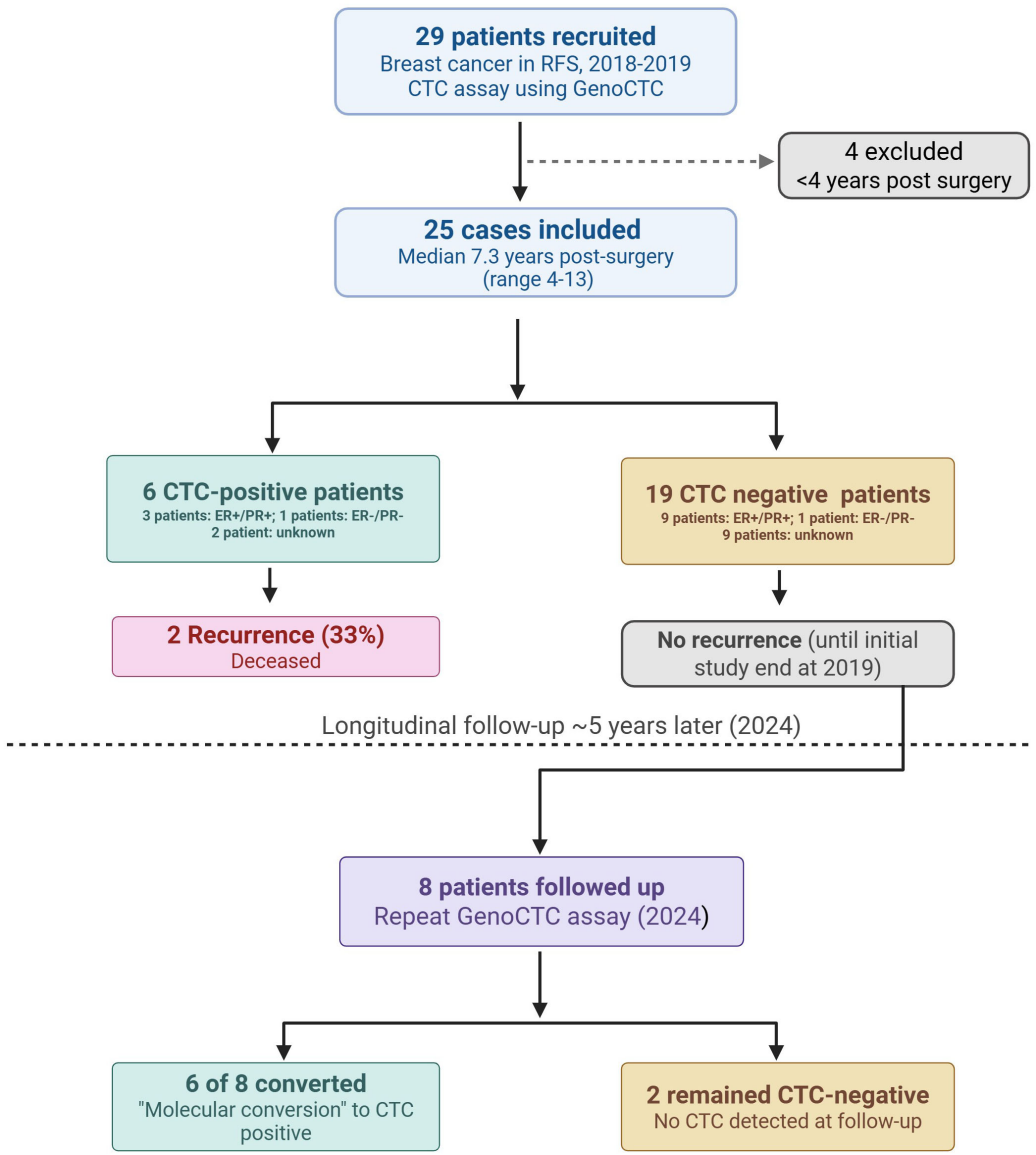
Study design and patient flow: Schematic representation of the study design describing the patient recruitment and the follow-up study. RFS, recurrence free survival; CTC, circulating tumor cell; ER, estrogen receptor; PR, Progesterone receptor.

**Figure 2:**
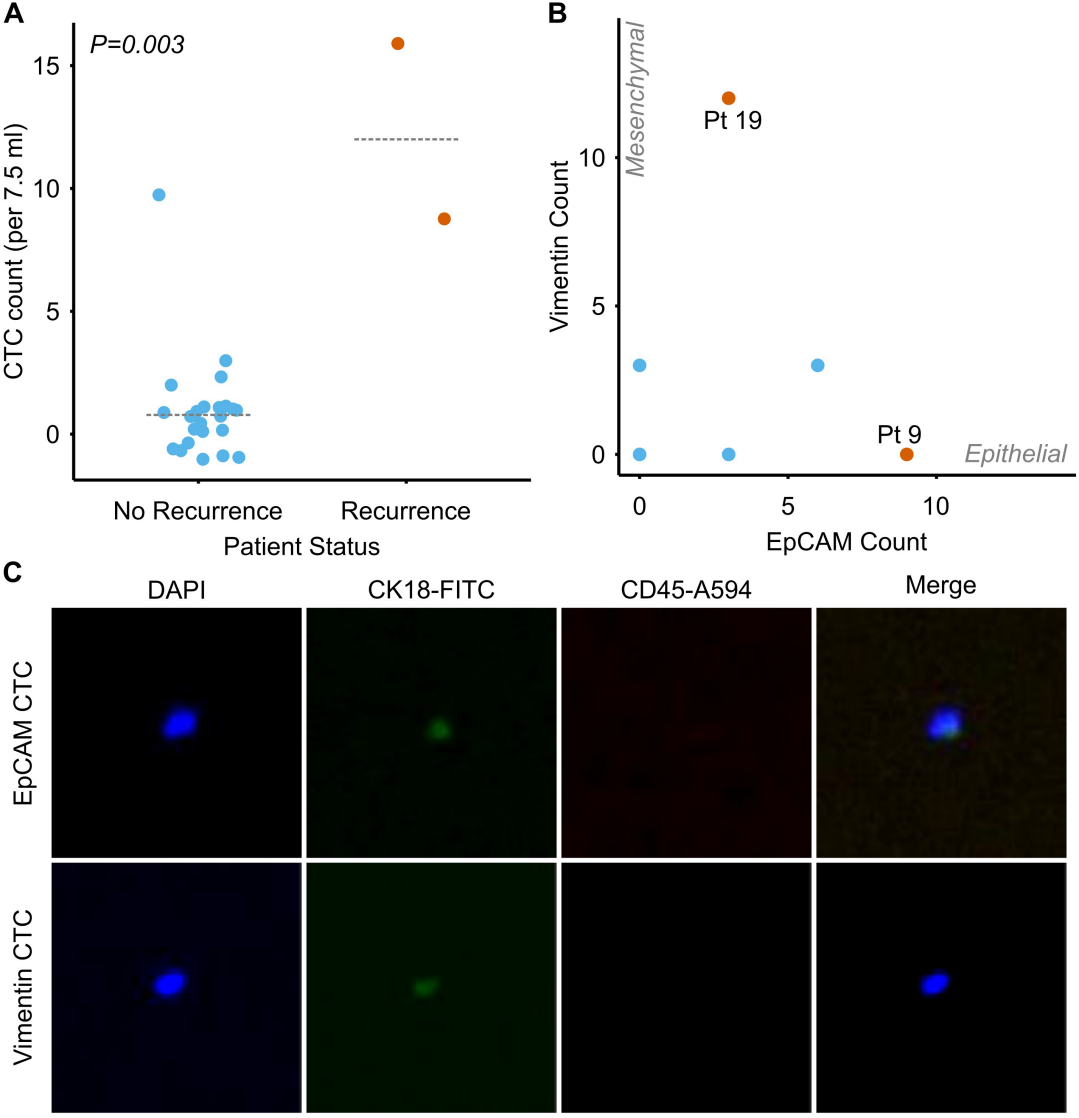
Baseline circulating tumor cell (CTC) burden and phenotypic composition as predictors of late recurrence. **(A)** Scatter plot illustrating total baseline CTC counts (normalized to 7.5 mL of blood) in the study cohort (N = 25), stratified by clinical outcome. Patients who experienced distant recurrence (n=2) had a significantly higher median CTC burden compared to those who remained clinically dormant (12.0 vs. 0.0 cells; P = 0.003, Mann-Whitney U test). Horizontal gray dotted lines represent the median CTC burden for each group. Relapses were uniquely associated with a high systemic CTC burden. **(B)** Phenotypic distribution map illustrating the relationship between EpCAM (x-axis) and Vimentin (y-axis) CTC counts at baseline for positive patients. Individual dots represent patients, with Patient (Pt) 9 exhibiting an epithelial-dominant recurrence pattern and Patient (Pt) 19 exhibiting a mesenchymal-dominant recurrence pattern. Patients displaying low level, indolent shedding of either phenotype remained clinically recurrence-free during the study period. **(C)** Representative immunofluorescence images of CTCs detected at baseline. Upper panel: CTC captured via anti-EpCAM enrichment. Lower panel: CTC captured via anti-Vimentin enrichment. Columns display channels for the nucleus (DAPI, blue), tumor marker Cytokeratin (FITC, green), leukocyte marker CD45 (A594, red), and the composite merged image. Cells are identified as CTCs based on the DAPI+/CK+/CD45- criteria.

Among the 25 patients (median 7.3 years post-surgery), baseline CTCs were detected in 24% (6/25) patients using EpCAM and Vimentin enrichment (**Figure 1**). The total CTC counts in positive patients ranged from 3 to 15 cells per 7.5 mL of blood (**Figure 2A**). Patient characteristics are summarized in **Table 1**.

**Table 1:** Clinicopathological characteristics and correlation with circulating tumor cell (CTC) burden in late-stage breast cancer survivors.

Characteristic	Total (N = 25)	CTC-negative (N = 19)	CTC-positive (N = 6)	P value
Age, years (mean)	61.9	62.4	60.5	0.774
Tumor stage, n (%)				0.289
Stage I	9 (36%)	5 (26%)	4 (67%)	
Stage II	7 (28%)	5 (26%)	2 (33%)	
Stage III/IV	5 (20%)	5 (26%)	0 (0%)	
Unknown	4 (16%)	4 (21%)	0 (0%)	
Receptor status, n (%)				
ER positive	12 (48%)	9 (47%)	3 (50%)	0.621
PR positive	11 (44%)	8 (42%)	3 (50%)	0.814
HER2 positive	6 (24%)	4 (21%)	2 (33%)	0.408
Clinical outcome, n (%)				0.0001
Recurrence	2 (8%)	0 (0%)	2 (33.3%)	

ER, estrogen receptor; PR, progesterone receptor; HER2, human epidermal growth factor receptor 2. P-values for categorical variables calculated by Fisher’s exact test; for age by independent samples t-test.

No statistically significant associations were found between baseline CTC positivity and age, tumor histology, tumor stage, or nodal involvement. However, CTC presence at baseline showed borderline association with recurrence (P = 0.05; 2 of the 6 CTC-positive patients recurred vs 0 of the 19 CTC negative patients; Fisher’s exact test) and significant correlation with mortality (P = 0.0016; Fisher’s exact test). Patients who experienced distant recurrence (n = 2) exhibited a significantly higher median baseline CTC count (12.0 vs. 0.0 cells/7.5 ml; P = 0.003; Mann–Whitney U test; **Figure 2A**). Notably, patients with clinical recurrence exhibited a high systemic burden (range: 9–15 cells), whereas patients with low or undetectable CTC burden (0–4 CTCs/7.5 ml) remained clinically dormant during the study period (**Figure 2A**). This suggests that while sporadic, low level CTC shedding may occur during stable dormancy, whereas a quantitative increase in systemic burden is associated with imminent clinical relapse.

### Phenotypic stratification of baseline CTCs

Baseline risk was further stratified by CTC phenotype to evaluate the biological nature of dormancy escape. Divergent phenotypes were observed in recurrence cases (**Figure 2B**). Patient 9 exhibited epithelial dominance (9 EpCAM+/CK+ cells), while Patient 19 showed a hybrid CK+/Vimentin+ phenotype (**Figure 2C**), indicative of active Epithelial-Mesenchymal Transition (EMT), where cells retain epithelial identity while acquiring the mesenchymal machinery necessary for systemic dissemination. Low-level mesenchymal shedding was observed in three patients who remained recurrence-free during the initial follow-up.

Among the CTC-positive patients, three (50%) were ER-positive and PR positive, two (33.3%) were HER2-positive. Among CTC positive patients, EGFR status was evaluable in 4 of 6, of whom one (25%) was EGFR positive. No significant correlation was observed between the presence of CTCs and marker status, likely reflecting the limited sample size of this feasibility cohort.

### Correlation between CTC burden and late recurrence probability

To quantify the predictive value of CTC detection, Pearson’s correlation analysis was performed between CTC burden and clinical recurrence. Out of the 6 CTC-positive cases, 2 (33.3%) subsequently developed clinically detectable recurrent disease and died. Both patients with a positive CTC assay were referred to a confirmatory diagnostic work up within 90 days of the initial CTC testing. Consecutively clinical recurrence was confirmed prior to or within this period-specifically 69 days after the positive CTC test in patient 9, and 43 days in patient 19. A strong significant correlation was observed between total CTC count and late recurrence (r=0.82; P = 7.87x 10^-8;^ **Table 2**). Sub group analysis confirmed significant correlations for both Ep+ CTCs and Vim+ CTCs individually, demonstrating that both epithelial and mesenchymal CTCs individually associated with recurrence risk (**Table 2**).

**Table 2:** Correlation between CTC burden and late recurrence.

Variable	Pearson correlation coefficient (p-value)
Total CTC vs. recurrence	0.82 (7.87 × 10^-8^)*
EpCAM+ CTC vs. recurrence	0.71 (2.75 × 10^-5^)*
Vimentin+ CTC vs. recurrence	0.62 (4.53 × 10^-4^)*

* indicated p-value less than 0.01.

### Longitudinal molecular conversion and dormancy exit

**Figure 3:**
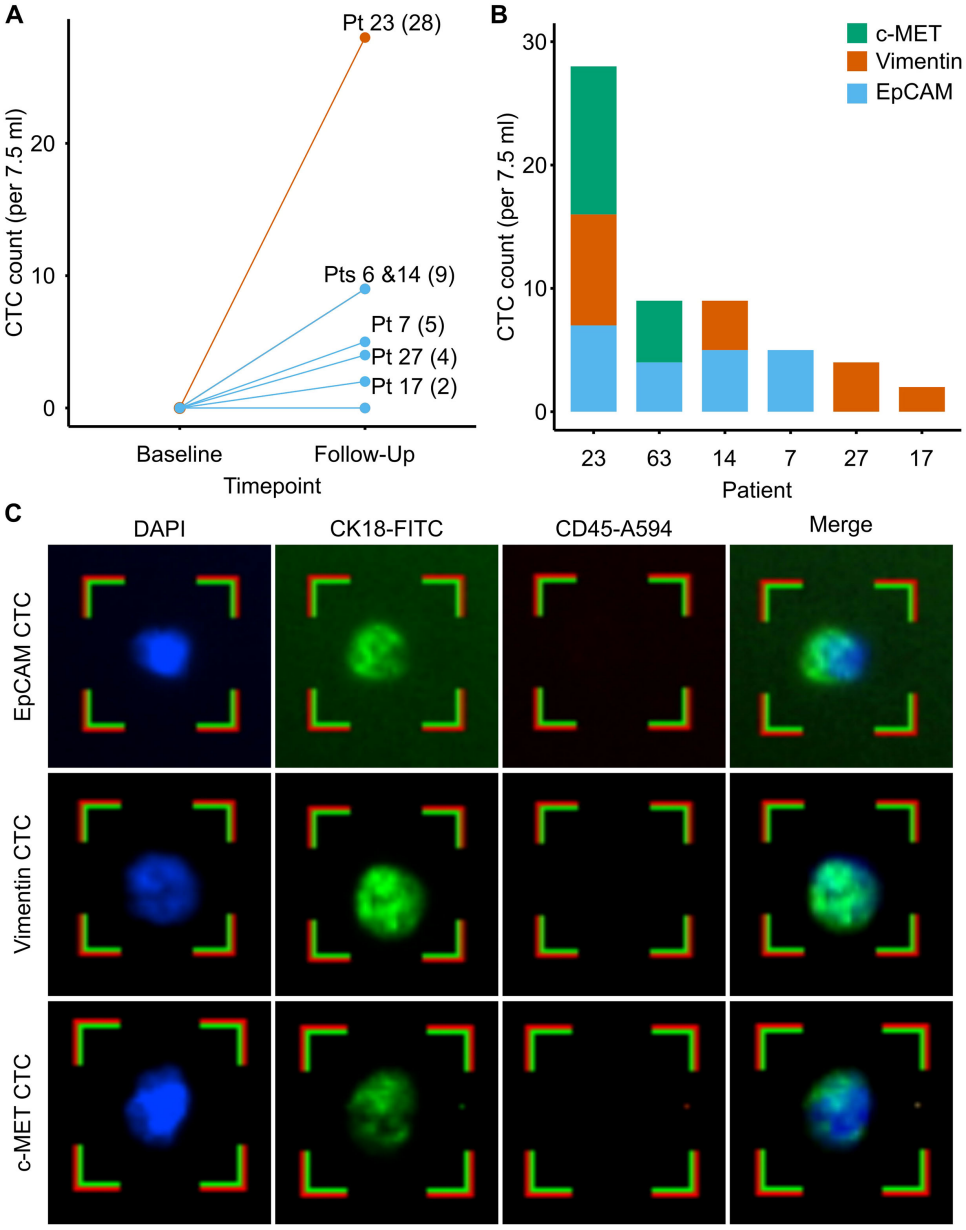
Longitudinal Emergence of c-MET-positive and vimentin-positive circulating tumor cells (CTCs) marks the exit from clinical dormancy. **(A)** Paired plot showing longitudinal changes in total CTC burden for eight patients who were initially CTC-negative at baseline. Data points represent CTC counts at baseline and at the median 5-year follow-up. Six of the eight patients (75%) demonstrated “molecular conversion,” defined as a shift from a negative to a positive CTC status. **(B)** Phenotypic composition of CTCs in the six patients demonstrating “molecular conversion” at follow-up. Stacked bars represent the total CTC burden for each patient, stratified by enrichment marker (EpCAM, Vimentin, and c-MET). Patient 23 exhibits a pronounced systemic “molecular burst” (total n=28), characterized by the expression of aggressive hybrid CK+/Vimentin+ (n=9) and c-MET+ (n=12) populations. **(C)** Representative immunofluorescence panels of the heterogeneous CTC populations emerging at follow-up. Rows display CTCs isolated via anti-EpCAM, anti-Vimentin, and anti-c-MET enrichment strategies, respectively. Staining confirms that all distinct phenotypic subpopulations retain epithelial identity (CK-FITC positive) and are distinct from leukocytes (CD45-A594 negative). The identification of c-MET–enriched CTCs (bottom row) confirms the acquisition of bypass signaling receptors on CK+ tumor cells.

To investigate the dynamic evolution of systemic metastatic risk, eight patients who were initially CTC-negative underwent longitudinal surveillance at a median follow up interval of 5-years (**Figure 3A**). Six of these eight patients (75%) demonstrated “molecular conversion,” transitioning from a CTC-negative baseline to a CTC-positive status at follow-up. The emergence of these cells was characterized by a dramatic increase in phenotypic complexity and the emergence of markers associated with poor prognosis in the metastatic setting (**Figure 3B**). Among the converters, hybrid CK+/Vimentin+ or CK+/c-MET+ were identified in 5 of the 6 patients (83%), indicating that dormancy exit is characterized by a high-degree of epithelial-mesenchymal plasticity rather than a simple epithelial phenotype. Notably, Patient 23, who remains clinically asymptomatic, exhibited a systemic “burst” of 28 CTCs consisting of hybrid CK+/Vim+ (n=9) and c-MET+ (n=12) populations, alongside purely epithelial CTCs (**Figure 3B, C**). At the time of this analysis, clinical follow-up for the converter cohort remained short relative to previously reported latencies between CTC detection and recurrence. The presence of c-MET+ CTCs in 2 of the 6 converters may reflect a bypass signaling mechanism that enables evasion of endocrine control (21). Representative immunofluorescence images confirming CTC identity (DAPI+/CK+/CD45-) across all three enrichment strategies are shown in **Figure 3C**.

## Discussion

This prospective feasibility study demonstrates that longitudinal, multi-marker CTC phenotyping can identify a biologically distinct pre-clinical event- herein termed “molecular conversion”- that may precede clinical late recurrence in breast cancer survivors. Using a dual biomarker enrichment strategy (Ep and Vim) at baseline and an expanded panel (Ep, Vim, and cMET) at follow-up, we demonstrate two key findings: (i) CTC burden at RFS (4–13 years after diagnosis) is a strong predictor of recurrence and CTC positivity showed borderline association with recurrence and mortality in long-term survivors; and (iii) the majority of the initially CTC-negative patients developed phenotypically complex, multi-marker CTC populations-including hybrid epithelial-mesenchymal and cMET positive cells-over a 5 years surveillance interval, without a concurrent clinical diagnosis of relapse.

The CTC-positivity rate observed in our cohort (24%), exceeds 4.8% reported in the landmark E5103 sub-study (14). The higher sensitivity of our study likely reflects dual Ep/Vim enrichment strategy which captures EMT-positive CTC sub-population, systemically missed by EpCAM-only methods. EMT-positive CTCs are consistently associated with worse prognosis in breast cancer, and their detection in the dormancy window may have fundamentally different clinical implications than detection of epithelial CTCs alone. However, it is important to note the very small sample size in our study, and any definitive conclusions regarding a higher detection rate would require confirmation in larger cohort studies.

The longitudinal finding of “molecular conversion” represents the most novel and clinically consequential observation of this study. The emergence of phenotypically complex CTCs in initially CTC-negative patients over a five-year interval, in the absence of any concurrent clinical diagnosis of relapse, suggests that systemic dissemination and phenotypic diversification may precede clinical relapse, by a potentially actionable time window. As of the December 2024 outcome cut-off, none of the 6 molecular converters had developed clinical or radiographic recurrence. The observation window following the follow-up CTC assay ranged from approximately 4 to 8 months across the converter cohort. This remains within the lower range of latencies reported between CTC positivity and clinical recurrence by Sparano et al (14), where lead time ranges from 0.1 to 2.8 years among CTC-positive patients who ultimately recurred. These findings raise the possibility that late recurrence in breast cancer reflects a biologically driven transition. However, continued longitudinal follow-up of the converter cohort is essential to determine the clinical trajectory and predictive value of this phenotypic transition.

The prominent role of epithelial-mesenchymal plasticity (EMP) is particularly noteworthy. Unlike classical EMT, which involves a complete loss of epithelial identity, EMP describes a spectrum of reversible hybrid states in which cells retain CK expression while co-expressing mesenchymal markers such as Vimentin (22). These hybrid CK+/Vim+ cells were detected in 66.7% of patients in the patients who converted from CTC-negative to CTC-positive status, suggesting that EMP-rather than a complete mesenchymal transition, is the predominant mechanism driving dormancy exit. Hybrid EMP cells have been shown to exhibit enhanced stemness, chemoresistance, and metastatic colonization efficiency compared to purely epithelial or mesenchymal populations (22–24), making their detection a clinically significant indicator of imminent aggressive relapse.

While mesenchymal markers in CTCs are established static indicators of poor prognosis in active disease (15, 16, 25, 26), our data uniquely highlight their potential role as a dynamic evolutionary switch marking the exit from dormancy. C-MET, the hepatocyte growth factor (HGF) receptor, is a known mediator of acquired resistance to anti-estrogen therapy in HR+ breast cancer, enabling tumor cells to activate proliferative and survival signaling independently of estrogen receptor stimulation (21, 27, 28). The presence of c-MET+ and Vimentin+ phenotypes may offer a possible mechanistic explanation for how dormant clones escape endocrine control. While c-MET was not assessed at baseline, its inclusion in the follow-up assay was prompted by prior evidences, including ours, identifying c-MET+ CTCs as independent predictors of poor prognosis in HR+ metastatic disease (19, 27, 29). The presence of c-MET+ CTCs in patients like patient 23, following years of clinical stability, suggests a possible “bypass” signaling mechanism where cells circumvent established endocrine pathways. This provides a biological rationale for investigating c-MET inhibitors in asymptomatic, CTC-positive patients to intercept metastasis before it becomes radiographically apparent.

The quantitative threshold of CTC burden appears to be clinically relevant. Patients with high systemic CTC counts (>9 CTCs/7.5 mL) experienced recurrence, while patients with lower CTC counts (0–4 cells/7.5 mL) remained clinically dormant. This suggests that, beyond binary CTC positivity, absolute CTC burden; and particularly the phenotypic composition of that burden-may provide prognostic information. A similar threshold dependent effect has been reported for CellSearch-based CTC detection in recurrence free survival setting; where recurrence occurred in 4.8% of patients with 0 CTCs, 16.7% of patients with 1 CTC, and 83.3% of patients with more than 2 CTCs in 7.5 mL of blood (14). Future studies should prospectively investigate whether quantitative and phenotypic CTC thresholds can guide therapeutic decision making in the long-term follow-up setting.

Several limitations of this study must be acknowledged. First, sample size is modest, as befitting a feasibility/proof-of-concept study, and our findings require validation in larger, prospectively designed cohorts with extended follow-up. Second, the clinical trajectory of the “molecular converters”, particularly Patient 23 with the high-burden CTC burst, remains an active follow-up question. The follow up period in our study is limited (approximately 4–8 months across the converter cohort), and none of the converters had an overt recurrence during this interval. However, it is worth noting that the follow-up period is within the lower range of previously reported latency period between CTC detection and clinical recurrence. Third, the absence of complete treatment history and molecular receptor data of all patients-resulting from the outpatient, non-oncology clinic setting of sample collection, limits a comprehensive multivariate risk analysis integrating therapy adherence, menopausal status, and extended endocrine therapy duration. Fourth, the parallel-processing nature of the GenoCTC^®^ platform, which analyzes separate blood aliquots for each biomarker, means that total systemic CTC burden may be underestimated compared to a single tube multi-marker assay. Fifth, the baseline assay and the follow-up assay used different blood collection tubes, and different imaging platforms, which could have introduced technical variability in the analyzed results. Though prior works have shown that intact tumor cell recovery from whole blood is not significantly affected by anticoagulant (EDTA)/preservative (Streck) tube type, when stored at prescribed temperature and processed within a defined stability window, technical variability introduced due to this cannot be completely ruled off (30). Sixth, the CTC enrichment panel used in the baseline and follow-up study were different. The follow-up study introduced cMET as an additional marker. The rationale for adding cMET as an additional CTC-enrichment marker is based on the emerging role of cMET as a driving factor of resistance, specifically endocrine resistance in breast cancer, as well as our own published study which identified cMET CTC as a significant independent predictor of prognosis in metastatic breast cancer. However, this asymmetry means, the cMET status at baseline is unknown and its apparent “emergence” at follow-up cannot be distinguished from pre-existing unmeasured cMET+ cells. Consequently, the proposed role of cMET as an indication of bypass signaling mechanism remains a hypothesis and requires validation. Seventh, this study did not differentiate pre-menopausal from post-menopausal women, a clinically relevant distinction given differential responses to extended adjuvant therapy. Future studies should address these limitations through multiple-center designs with prospective data collection, standardized treatment protocols, and complete molecular characterization of primary and recurrent tumors. Additionally, high-resolution mapping of the genomic and transcriptomic variance within emerging CTC populations by single-cell RNA sequencing (scRNA-seq) could enable the identification of precise molecular signatures that trigger the transition from indolent dormancy to aggressive relapse.

Despite these limitations, the present findings have important implications for clinical practice and future research. The identification of molecular recurrence preceeding clinical relapse offers a rationale for investigating a paradigm shift in post-treatment surveillance; from the current radiologic imaging detection toward longitudinal liquid biopsy monitoring. Patients who convert to a CTC-positive status with phenotypically aggressive profiles may represent a distinct risk category warranting closer surveillance; whether this should prompt therapeutic intervention, such as, re-engagement with endocrine therapy, or enrollment in interventional clinical trials, remains an important question for future prospective studies. If validated, integrating liquid biopsy surveillance into long-term follow up could offer a less invasive, more proactive alternative to current imaging-based models.

In summary, this prospective feasibility study demonstrates that longitudinal monitoring of CTC phenotypic composition could provide a dynamic, real-time window into the biology of breast cancer dormancy and its exit. The absolute CTC counts may be a crude metric; the specific detection of mesenchymal (Vimentin) and growth-factor (c-MET) markers —particularly within CK+ hybrid populations—may help distinguish between stable dormancy from a higher-risk population, though this needs to be validated with a longer clinical follow-up study. Our data suggests that phenotypic “conversion” in the bloodstream may represent an early correlate of late recurrence. These findings support further prospective investigation of multimodal CTC assays as a potential tool into the standard follow-up care for long-term survivors, with the goal of enabling earlier therapeutic interception of metastatic awakening of breast cancer.

## Data Availability

The original contributions presented in the study are included in the article/supplementary material. Further inquiries can be directed to the corresponding authors.

## References

[1] Siegel RL Giaquinto AN Jemal A . Cancer statistics, 2024. CA Cancer J Clin. (2024) 74:12–49. doi: 10.3322/caac.21820 38230766

[2] Pan H Gray R Braybrooke J Davies C Taylor C McGale P . 20-year risks of breast-cancer recurrence after stopping endocrine therapy at 5 years. N Engl J Med. (2017) 377:1836–46. doi: 10.1056/NEJMoa1701830 29117498 PMC5734609

[3] Pedersen RN Esen BO Mellemkjaer L Christiansen P Ejlertsen B Lash TL . The incidence of breast cancer recurrence 10-32 years after primary diagnosis. J Natl Cancer Inst. (2022) 114:391–9. doi: 10.1093/jnci/djab202 34747484 PMC8902439

[4] Early Breast Cancer Trialists’ Collaborative Group . Reductions in recurrence in women with early breast cancer entering clinical trials between 1990 and 2009: a pooled analysis of 155 746 women in 151 trials. Lancet. (2024) 404:1407–18. doi: 10.1016/S0140-6736(24)01745-8 39396348 PMC12979726

[5] Dogan I Aydin E Khanmammadov N Paksoy N Ferhatoglu F Ak N . Long-term outcomes and predictors of recurrence in node-negative early stage breast cancer patients. J Cancer Res Clin Oncol. (2023) 149:14833–41. doi: 10.1007/s00432-023-05276-y 37594533 PMC11796621

[6] van't Veer LJ Meershoek-Klein Kranenbarg E Dujim-de Carpentier M Van de Velde CJH Kleijn M Dreezen C . Selection of patients with early-stage breast cancer for extended endocrine therapy: a secondary analysis of the IDEAL randomized clinical trial. JAMA Netw Open. (2024) 7:e2447530. doi: 10.1001/jamanetworkopen.2024.47530 39602119

[7] Sparano JA Crager M Gray RJ Tang G Hoag J Baehner F . Clinical and genomic risk for late breast cancer recurrence and survival. NEJM Evidence. (2024) 3:EVIDoa2300267. doi: 10.1056/EVIDoa2300267 39041867 PMC11730053

[8] Sgroi DC Sestak I Cuzick J Zhang YI Schnabel CA Schroeder B . Prediction of late distant recurrence in patients with oestrogen-receptor-positive breast cancer: a prospective comparison of the breast-cancer index (BCI) assay, 21-gene recurrence score, and IHC4 in the TransATAC study population. Lancet Oncol. (2013) 14:1067–76. doi: 10.1016/S1470-2045(13)70387-5 24035531 PMC3918681

[9] Sestak I Dowsett M Zabaglo L Lopes-Knowles E Sean F Cowens JW . Factors predicting late recurrence for estrogen receptor-positive breast cancer. J Natl Cancer Inst. (2013) 105:1504–11. doi: 10.1093/jnci/djt244 24029245 PMC3787911

[10] Dowsett M Sestak I Regan MM Dodson A Giuseppe V Thurlimann B . Integration of clinical variables for the prediction of late distant recurrence in patients with estrogen receptor–positive breast cancer treated with 5 years of endocrine therapy: CTS5. J Clin Oncol. (2018) 36:1941–8. doi: 10.1200/JCO.2017.76.4258 29676944 PMC6049399

[11] Chelakkot C Yang H Shin YK . Relevance of circulating tumor cells as predictive markers for cancer incidence and relapse. Pharm (Basel). (2022) 15. doi: 10.3390/ph15010075 35056131 PMC8781286

[12] Cristofanilli M Budd GT Ellis MJ . Circulating tumor cells, disease progression, and survival in metastatic breast cancer. N Engl J Med. (2004) 351:781–91. doi: 10.1056/NEJMoa040766 15317891

[13] Pierga J-Y Hajage D Bachelot T Delaloge S Brian E Campone M . High independent prognostic and predictive value of circulating tumor cells compared with serum tumor markers in a large prospective trial in first-line chemotherapy for metastatic breast cancer patients. Ann Oncol. (2012) 23:618–24. doi: 10.1093/annonc/mdr263 21642515

[14] Sparano J O'Neill A Alpaugh K Wolf AC Northfelt DW Dang CT . Association of circulating tumor cells with late recurrence of estrogen receptor–positive breast cancer. JAMA Oncol. (2018) 4:1700. doi: 10.1001/jamaoncol.2018.2574 30054636 PMC6385891

[15] Bulfoni M Gerratana L Del Ben F Marzinotto S Sorrentino M Turetta M . In patients with metastatic breast cancer the identification of circulating tumor cells in epithelial-to-mesenchymal transition is associated with a poor prognosis. Breast Cancer Res. (2016) 18:30. doi: 10.1186/s13058-016-0687-3 26961140 PMC4784394

[16] Papadaki MA Stoupis G Theodoropoulos PA Mavroudis D Georgoulias V Agelaki S . Circulating tumor cells with stemness and epithelial-to-mesenchymal transition features are chemoresistant and predictive of poor outcome in metastatic breast cancer. Mol Cancer Ther. (2019) 18:437–47. doi: 10.1158/1535-7163.MCT-18-0584 30401696

[17] Satelli A Mitra A Brownlee Z Xia X Belloister S Overman ML . Epithelial-mesenchymal transitioned circulating tumor cells capture for detecting tumor progression. Clin Cancer Res. (2015) 21:899–906. doi: 10.1158/1078-0432.CCR-14-0894 25516888 PMC4334736

[18] Wang Y Liu Y Zhang L Tong L Gao Y Hu F . Vimentin expression in circulating tumor cells (CTCs) associated with liver metastases predicts poor progression-free survival in patients with advanced lung cancer. J Cancer Res Clin Oncol. (2019) 145:2911–20. doi: 10.1007/s00432-019-03040-9 31646374 PMC6861204

[19] Park J Chang ES Kim JY Chelakkot C Sung M Song JY . c-MET-positive circulating tumor cells and cell-free DNA as independent prognostic factors in hormone receptor-positive/HER2-negative metastatic breast cancer. Breast Cancer Res. (2024) 26:13. doi: 10.1186/s13058-024-01768-y 38238761 PMC10797795

[20] Chelakkot C Ryu J Kim MY Kim JS Kim D Hwang J . An immune-magnetophoretic device for the selective and precise enrichment of circulating tumor cells from whole blood. Micromachines (Basel). (2020) 11. doi: 10.3390/mi11060560 32486306 PMC7345362

[21] Lai X Zhang Y Li M Yu S Wang S Zhang S . HGF/c-Met promotes breast cancer tamoxifen resistance through the EZH2/HOTAIR-miR-141/200a feedback signaling pathway. Mol Carcinog. (2025) 64:769–83. doi: 10.1002/mc.23878 39853766

[22] Augimeri G Gonzales ME Paoli A Eido A Choi Y Burman B . A hybrid breast cancer/mesenchymal stem cell population enhances chemoresistance and metastasis. JCI Insight. (2023) 8. doi: 10.1172/jci.insight.164216 37607007 PMC10561721

[23] Liao N Guo Q Chen J Tan F Huang Y Yi J . Evaluating metastatic risk in breast cancer through CTCs and L1CAM expression. Front Oncol. (2025) 15. doi: 10.3389/fonc.2025.1686166 41306535 PMC12643865

[24] Ma L Wang Y Qui S Shi M Tang W Hu H . Circulating tumor cells in breast cancer bone metastasis: mechanisms, clinical relevance, and future directions. Cell Death Discov. (2025) 12:48. doi: 10.1038/s41420-025-02910-1 41372129 PMC12830773

[25] Guan X Ma F Li C Wu X Hu S Huang J . The prognostic and therapeutic implications of circulating tumor cell phenotype detection based on epithelial-mesenchymal transition markers in the first-line chemotherapy of HER2-negative metastatic breast cancer. Cancer Commun (Lond). (2019) 39:1. doi: 10.1186/s40880-018-0346-4 30606259 PMC6319003

[26] Guan X Li C Li Y Wang J Yi Z Liu B . Epithelial-mesenchymal-transition-like circulating tumor cell-associated white blood cell clusters as a prognostic biomarker in HR-positive/HER2-negative metastatic breast cancer. Front Oncol. (2021) 11:602222. doi: 10.3389/fonc.2021.602222 34150608 PMC8208036

[27] Iovino F Diana A Carlino F Ferraraccio F Antoniol G Fisone F . Expression of c-MET in estrogen receptor positive and HER2 negative resected breast cancer correlated with a poor prognosis. J Clin Med. (2022) 11. doi: 10.3390/jcm11236987 36498560 PMC9738605

[28] Raghav KP Wang W Liu S Chavez-MacGregor M Meng X Hortobagyi GN . cMET and phospho-cMET protein levels in breast cancers and survival outcomes. Clin Cancer Res. (2012) 18:2269–77. doi: 10.1158/1078-0432.CCR-11-2830 22374333 PMC3821167

[29] Ilie M Szafer-Glusman E Hofman V Long-Mira E Suttmann R Darbonne W . Expression of MET in circulating tumor cells correlates with expression in tumor tissue from advanced-stage lung cancer patients. Oncotarget. (2017) 8:26112–21. doi: 10.18632/oncotarget.15345 28212540 PMC5432243

[30] Ilie M Szafer-Glusman E Hofman V Long-Mira E Suttmann R Darbonne W . CTC-mRNA (AR-V7) analysis from blood samples—impact of blood collection tube and storage time. Int J Mol Sci. (2017) 18:1047. doi: 10.3390/ijms18051047 28498319 PMC5454959

